# Associations between community engagement, human flourishing, and mental illness symptoms among college students

**DOI:** 10.1371/journal.pmen.0000690

**Published:** 2026-08-17

**Authors:** Sydney Clark, Steven Byungkwan Ko, Blake L. Jones, AliceAnn Crandall, Carl Lee Hanson

**Affiliations:** 1 Department of Public Health, Brigham Young University, Provo, Utah, United States of America; 2 Department of Psychology, Brigham Young University, Provo, Utah, United States of America; Affiliated Guangji Hospital of Soochow University, CHINA

## Abstract

Poor mental health, including anxiety and depression, is prevalent among college students. Identifying protective factors is essential for enhancing mental health. Previous research suggests engagement in various communities may be important for mental health. This study examined the associations between engagement in community domains (e.g., family, school, work, and church) on individual mental health outcomes among college students. Participants (*N* = 379) were recruited through a general education undergraduate introductory psychology course. Participants completed a survey measuring anxiety, depression, flourishing, and community engagement. Data were analyzed using structural equation modeling (SEM) in MPlus8. The SEM demonstrated good fit to the data (RMSEA = .049, CFI = .966). Engagement across family, peer, church, and school domains was positively associated with higher individual flourishing, with peer engagement showing the strongest association (*β* = 0.26, p < .05). Higher flourishing was associated with lower levels of depression and anxiety. Indirect associations between community engagement and mental health outcomes through flourishing were significant across all domains (*β* = -.09, p < .05). School engagement was also independently associate with anxiety symptoms. These findings highlight the importance of fostering engagement across family, school, peer, and church domains, which may enhance individual flourishing and reduce depression and anxiety among young adult college students.

## Introduction

Poor mental health among college students is a pressing issue. The Healthy Minds Survey reports that 38% of college students struggle with some form of depression, and 34% of students struggle with some form of anxiety [1]. College is a transitional period often marked by academic pressure, separation from family, and other stressors that frequently lead to students experiencing their first onset of mental health symptoms [2]. Various factors, such as identity confusion, financial hardship, relationship struggles, and educational demands, may also combine to create a particularly challenging time for many students [3].

A growing number of researcher and policy makers worldwide are focusing on the positive aspects of mental health rather than mental illness. The World Health Organization defines mental health as a state of well-being that allows individuals to handle the stresses of life, recognize their strengths, learn to work effectively, and actively participate in their community [4]. To expand on that, Westerhof and Keyes have further enhanced the definition of mental health by defining two terms: flourishing and languishing. Flourishing is defined as a state where a person experiences strong emotional well-being along with an optimal level of positive psychological and social functioning. In contrast, languishing is defined as a state marked with low emotional well-being and negative psychological and social functioning [5]. According to Wielemaker, a range of factors contribute to individual flourishing, such as physical activity, resilience, sleep quality, social connection, and loneliness [6].

Seligman’s PERMA model suggests that flourishing is obtained through five core components: positive emotions, engagement, relationships, meaning, and accomplishment [7,8]. These components represent antecedents to flourishing individuals that have been widely studied in positive psychology as a conceptual framework [9]. Within the framework of positive psychology, engagement plays an important role and is referred to as deep psychological involvement, immersion, and absorption in activities. This concept emerges from Flow Theory, which refers to complete absorption in what one is doing leading to a transformation in one’s sense of time [10]. It was originally conceptualized by Csikszentmihalyi [11]. High levels of engagement has been associated with individual flourishing and mental health in the workplace and has been defined in terms of vigor, dedication, and absorption [12–15]. Similarly, engagement has been shown to influence student achievement and school behavior [16].

Given that engagement represents a pathway to individual flourishing and mental health in worksite settings, it is important to explore these relationships across other ecological levels. Bronfenbrenner’s Ecological Systems Theory [17,18] purports that individual functioning is influenced by the multiple environments in which individuals are nested in such as family, school, peer groups (microsystems), their interactions (mesosystems), and broader community and societal contexts (exosystems and macrosystems). Engagement in leisure activities has also been associated with higher subjective well-being, positive emotion, and reduced stress [19]. Civic or community engagement, such as volunteering, has been shown to enhance mental health and life satisfaction [20,21].

Guided by social ecological and positive psychology frameworks, the purpose of this study was to assess how community engagement across ecological levels (e.g., family, school, work, church) dimensions of human flourishing, and symptoms of depression and anxiety. Human flourishing was assessed using a multidimensional measure that included both individual flourishing and financial/material stability [22]. This aim gave rise to three hypotheses: 1) Higher levels of community engagement would be positively associated with individual flourishing; 2) Higher levels of individual flourishing would be associated with lower levels of depression and anxiety; and 3) Individual flourishing would mediate the relationship between community engagement and symptoms of depression and anxiety. Financial/material stability was also explored as it is theoretically and important dimension of human flourishing.

## Methods

### Ethics statement

All procedures were approved by the Brigham Young University Institutional Review Board (IRB#: IRB2025–050). Informed consent was obtained using an implied (non-written) consent process administered electronically at the beginning of the survey. Participants were provided with information about the study purpose, procedures, and their rights, including the ability to withdraw at any time. All participants were at least 18 years of age.

### Sample

A survey was administered to college students of at least 18 years of age attending a private university in the intermountain west of the United States. They were enrolled in an introductory psychology course (PSYCH 111), which is a general education requirement and thus represents students from all different majors. Participants that consented to participate were screened for eligibility. A total of 401 students completed the survey, but 22 participants were missing information on key demographic factors included as controls and were omitted from the final analysis, resulting in a total of 379 participants.

### Procedures

The survey was administered online using the Qualtrics platform. Participants were recruited from an undergraduate introductory psychology course (PSYCH 111) at a private university in intermountain western United States between February 14, 2025 and May 6, 2025. Research assistants presented information about the study during class sessions and a QR code was provided to direct students to the online survey.

The survey took approximately 15 minutes to complete and consisted of 139 questions. Upon completion, participants received a $5 gift card and were also eligible to receive psychology course credit. Mental health resources were provided at the end of the survey.

### Measures

Human flourishing was measured using a 12-item scale created by Tyler VanderWeele with the Harvard Human Flourishing Program and includes domains related to psychological well-being, relationships, meaning and purpose, heath, and financial/material stability [22]. Each item was on a 10-point Likert scale (e.g., 0 = *not satisfied at all* to 10 = *completely satisfied*): Sample items included “Overall, how satisfied are you with life as a whole these days” and “My relationships are as satisfying as I would want them to be.” Exploratory factor analysis indicated that a two-factor structure best fit the data, with the 10 original human flourishing items loading on one factor (labeled individual flourishing) and the two financial and material stability items loading on the second factor (labeled financial and material stability). Because financial and material stability is considered an important domain within VanderWelle’s model of human flourishing [22], this factor was retained in the measurement model and examined as a potential pathway between community engagement and mental health outcomes.

Engagement was measured using the three-item Utrecht Work Engagement Scale (UWES-3) [23], adapted to assess engagement across multiple community domains, including peer, family, school, and church. This UWES-3 assesses three core dimensions of psychological engagement: vigor, dedication, and absorption. Although originally developed for occupational settings, the scale focuses on positive psychological states rather than any specific role or setting. Researchers have successfully used measures of vigor, dedication, and absorption as meaningful indicators of student engagement and well-being [24,25]. Given the central role of community involvement in both positive psychology and ecological frameworks, UWES-3 items were adapted by replacing any work-related language with domain-specific language (e.g., In my peer group, I feel bursting with energy). Participants rated each item on a 5-point Likert scale ranging from 0 (*strongly disagree*) to 4 (*strongly agree*).

Anxiety symptoms were measured using the Generalized Anxiety Disorder-7 (GAD-7) [26], a seven-item self-report measure of anxiety symptom frequency over the past two weeks. Items were rated on a 4-point Likert scale ranging from 0 (not at all) to 3 (nearly every day). Items were summed to create a total anxiety score. Consistent with clinical guidelines, scores of 10 or higher were used to indicate moderate-to-severe anxiety. For the analysis, a dichotomous variable was created to distinguish participants with moderate to severe anxiety from those with mild or no anxiety.

Depressive symptoms were measured using the Patient Health Questionnaire-9 (PHQ-9) [27], a nine-item self-report measure assessing depressive symptoms over the past two weeks based on diagnostic criteria for major depressive disorder. Items were rated on a 4-point Likert scale ranging from 0 (not at all) to 3 (nearly every day). Items were summed to create a total depression score, with higher scores indicating greater symptom severity. Consistent with clinical guidelines, scores of 10 or higher were used to indicate moderate to severe depressive symptoms. For the analysis, a dichotomous variable was created to distinguish participants with moderate to severe depression from those with mild or no depression.

Demographic controls included participant race, age (in years), sex, marital status, and affiliation with the predominant religion of the student body.

### Analysis

Data were cleaned and item distributions were analyzed in Stata version 18. A confirmatory factor analysis (CFA) was conducted in Mplus verion 8 to examine the measurement model (comprised of individual flourishing and engagement constructs). Items were required to have a factor loading > 0.30. Adequate model fit was defined as RMSEA < 0.08 and CFI 0.90. After confirming the measurement model, a structural equation model (SEM) was estimated by regressing moderate/severe depression and anxiety on individual flourishing and engagement. Individual flourishing was also regressed on peer, family, church, and school engagement. Demographic variables were controlled for by regressing all variables of interest (dependent, independent, and mediating variables) on the demographic variables. Mediation was tested using the Mplus “ind” command. To ensure robust standard errors for the indirect effects, 5,000 bootstraps were used. The same model fit criteria applied to the SEM model as to the CFA model. All models were estimated using the robust weighted least squares mean and variance adjusted (WLSMV) estimator, appropriate for categorical data.

## Results

### Descriptive statistics

Participant age ranged from 18-35 (*M* = 20.43, *SD* = 2.07) (see Table 1). Approximately 60% were female and 93% reported being white. Most of the sample (97%) reported being of members of the Church of Jesus Christ of Latter-Day Saints, and 8% reported being married. Based on the individual flourishing scale (range 0–10), the mean score was 7.00 (*SD* = 1.23). Approximately 24% of the sample was classified as having moderate to severe anxiety, and 27% was classified as having moderate to severe depression.

**Table 1:** Sample and Descriptive Statistics.

Variable	Mean (*SD*) or %
Demographics	
White (%)	93.40
Age, *M (SD)*	20.43 (2.07)
Female (%)	59.89
Church of Jesus Christ of Latter-Day Saint (%)	97.36
Married (%)	8.44
Engagement	
Peer, *M (SD)*	3.88 (0.88)
Family *M (SD)*	4.24 (0.89)
Church *M (SD)*	3.47 (1.13)
School *M (SD*)	3.89 (0.82)
Mental Health	
Individual Flourish, *M (SD)*	7.00 (1.23)
Anxiety (%)	23.60
Depression (%)	26.84

*Flourishing scale range 0–10.

** Range 1–5.

The mean engagement score among peer groups was 3.88 (*SD* = 0.88). Engagement at the family level had the highest mean at 4.24 (*SD* = 0.89). Engagement in church communities had a mean of 3.47 (*SD* = 1.13) and school communities had a mean of 3.89 (0.82).

### Confirmatory factor analysis

Model fit for the final measurement model was good (RMSEA = .049; CFI = 0.966). The CFA confirmed a 2-factor structure for individual flourishing, with factor loadings ranging from.33 to.88. We correlated error terms for items in each sub-domain of the general flourishing construct (e.g., the error terms from the two happiness and life satisfaction items were correlated; the error terms from the two items from the mental and physical health items were correlated; and so forth). Factor loadings for the four engagement constructs were also above minimum requirements (peer engagement: range = .74 to.93; family engagement: range = .86 to.95; church engagement: range = .89 to.96; school engagement: range = .76 to.85).

### Structural equation model (SEM)

**Fig 1:**
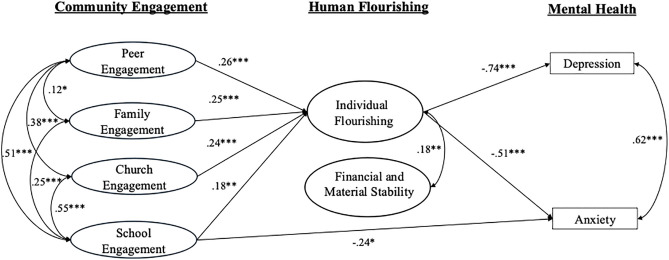
Path analysis examining community engagement, human flourishing and mental health, N = 379. *p < .05. **p < .01. ***p < .001. Model Fit: RMSEA = 0.049; CFI = 0.966.

The results of the study revealed that community engagement (e.g., peer engagement, family engagement, church engagement, and school engagement) was directly associated with higher individual flourishing scores. As shown in Fig 1 (Model Fit: RMSEA 0.049; CFI 0.966), engagement across all four ecological domains was associated with higher levels of individual flourishing though not with financial and material stability. Specifically, the domains family engagement, school engagement, church engagement, and peer engagement showed a direct positive path to individual flourishing scores (*p* < 0.05). Of the four, peer engagement had the strongest association with individual flourishing (*β* = 0.26). Higher individual flourishing scores were directly associated with decreased depression and anxiety symptoms. School engagement was the only ecological level measured that had a direct effect on reducing anxiety symptoms.

While financial and material stability was correlated with individual flourishing, it was not a mediating variable between community engagement and mental health.

Significant paths only are shown. Betas are standardized. Model controls for race, age, gender, religious affiliation, and marital status.

Results for the indirect effects (Table 2) indicated that individual flourishing mediated the association between all forms of community engagement and depression/anxiety. Higher levels of peer, family, church, and school engagement were each associated with lower depression and anxiety symptoms through individual flourishing. Indirect effects were consistently slightly stronger for depression than anxiety.

**Table 2:** Individual Flourishing as a mediator between community engagement and mental illness symptoms.

Path	*β*	Z-Score	P-value
Peer Engagement to Depression	-0.19	3.44	0.001
Family Engagement to Depression	-0.18	3.60	< 0.001
Church Engagement to Depression	-0.18	3.28	0.001
School Engagement to Depression	-0.14	2.55	0.011
Peer Engagement to Anxiety	-0.13	3.07	0.002
Family Engagement to Anxiety	-0.13	2.96	0.003
Church Engagement to Anxiety	-0.12	2.74	0.006
School Engagement to Anxiety	-0.09	2.31	0.021

## Discussion

The purpose of the study was to assess the relationships between community engagement, dimensions of human flourishing, and mental health outcomes within the context of positive psychology and social ecological (i.e., peer, family, church, school) frameworks. Overall, the results support the study hypothesis that community engagement is associated with higher levels of individual flourishing and is linked to lower levels of depression and anxiety. In addition, individual flourishing proved to be an important mediating factor through which community engagement was related to mental health outcomes, whereas financial/material stability did not.

Results of this study revealed that engagement across socioecological contexts was positively associated with individual flourishing. These findings align closely with research demonstrating that adolescent and young adult connectedness to peers, family, school, and faith-based communities is a cornerstone of mental health and well-being [28–30]. Specifically, peer engagement has been shown across reviews and intervention trials to be strongly associated with better wellbeing and resilience, functioning as a major protective factor during adolescence and young adulthood [28–30]. Similarly, family engagement and connectedness predict lower involvement in risk behaviors and stronger emotional health, and support later civic and religious engagement [30,31]. School engagement, particularly feeling connected to teachers, staff, and the broader school climate, is consistently linked to better mental health outcomes, fewer risk behaviors, and higher overall wellbeing [29,32]. Finally, church and religious engagement during adolescence have been associated with stronger identity development, increased prosocial behavior, and greater civic and community involvement in adulthood--outcomes linked to individual flourishing [31,33,34]. Given the religious composition of the current sample, church engagement may reflect more than religious participation alone. For example, engagement in church communities may also represent social integration, access to support networks, and shared cultural values that interact with student life. As such, these observed associations should not be interpreted solely because of religious participation but may also be due to broader social and community factors.

Higher levels of individual flourishing were associated with lower levels of depression and anxiety, supporting its important role as a protective factor for mental health. Several studies confirm the relationship between flourishing as a protector against mental disorders [35–38]. For example, Schotanus-Dijkstra et al. found that the risk of incident mood disorders was reduced by 28% and anxiety disorders by 53% when an individual was flourishing [39]. Individual flourishing may protect against adverse mental health outcomes by promoting positive emotions, meaning and purpose, and social integration and wellbeing [40]. These finding emphasize the important fact that mental health is more than the absence of mental illness and includes positive psychological functioning as a protection against psychopathology. Additionally, the present findings however extend prior research by highlighting the role of individual flourishing as it helps link larger social factors to mental health outcomes.

In the current study, individual flourishing served as a mediator between community engagement and mental health outcomes. Previous studies show a strong positive correlation between regular church attendance and improved mental health [22,41–43]. Other studies show the strength between school engagement and improved mental health [44]. Although there is less research regarding the relationship between peer group engagement and mental health, researchers have found that peer support plays an important role in adolescents with mental health needs [45,46]. Similarly, the relationship between family engagement and mental health has been shown through examining the impact of family engagement on treating mental illness [47,48]. Notably, the current findings demonstrate that engagement in all four domains is significantly associated with improved mental health outcomes.

Financial and material stability was also studied because it represents an important domain within VanderWeel’s model of human flourishing [22]. Although results revealed it was a distinct factor independent from individual flourishing, it was not a significant mediator between community engagement and mental health outcomes. One possible explanation is the sample included traditional colleges students that are in similar financial situations than more heterogeneous adult populations.

### Limitations

These results should be interpreted in the context of the following limitations. First, this study is a cross-sectional design that does not allow for any causal conclusions or ordering of the observed associations. Although the hypothesized model was informed by theoretical frameworks, other explanations are possible. For example, students with higher levels of individual flourishing or fewer mental health symptoms may be more likely to engage in community domains. Additionally, unmeasured factors such as personality characteristics, social support, or family functioning may influence community engagement and mental health. Future research using longitudinal or experimental designs could help establish causality and directionality of associations. Second, the sample was drawn from a single university using a convenience sample of students enrolled in an introductory psychology course. While this course fulfills a general education requirement and includes students from a variety of majors, participants may not be representative of the broader college student population. Students who chose to participate in the study may differ from nonparticipants in their interest in mental health, thus introducing selection bias. Additionally, the sample was relatively homogeneous in that racial/ethnic and religious diversity was limited, which may limit the generalizability of the findings. Future studies should examine these relationships using more diverse and representative samples across multiple educational settings. Third, although the UWES-3 was adapted to assess engagement across family, peer, school, and church contexts, the scale was originally developed for occupational settings. While the scale has acceptable psychometric properties in the present sample, additional research is needed to further validate this scale across community domains. Finally, while individual flourishing helps to explain some of the association between community engagement and mental health outcomes, the presence of a significant direct effect from school engagement to anxiety suggests partial meditation, and that factors beyond individual flourishing may help explain how community engagement influences mental health outcomes. Future research should examine additional mechanisms that may also explain how community engagement influences mental health such as coping strategies, individual stressors, and environmental/institutional factors.

## Conclusion

This study explored associations between engagement across various community domains, individual flourishing, and mental health among college students. Findings indicated that greater engagement in family, peer, church, and school settings was associated with increased individual flourishing, which in turn was associated with lower symptoms of depression and anxiety. Individual flourishing appeared to play a central role in linking community engagement domains with mental health outcomes, although school engagement also showed a direct relationship with anxiety. These findings emphasize the importance of community engagement and well-being in understanding mental health among college students.
