# Supplementary material for: Associations between community engagement, human flourishing, and mental illness symptoms among college students
Source: PLOS Ment Health. 2026 Aug 17;3(8):e0000690. doi: 10.1371/journal.pmen.0000690 (PMC13480599; doi:10.1371/journal.pmen.0000690)
Supplement: Supplementary file 1 [file pmen.0000690.s002.docx]

Dear Editor,

Thank you for the opportunity to revise our manuscript entitled **"Associations between Community Engagement, Human Flourishing, and Mental Illness Symptoms among College Students."** We appreciate the thoughtful and constructive comments provided by you and the reviewers, which have substantially strengthened the manuscript.

We have carefully considered each comment and revised the manuscript accordingly. A detailed point-by-point response is provided in the table below. The revised manuscript with tracked changes has also been included for your review.

We believe these revisions have improved the rigor, clarity, and interpretation of our findings. We appreciate the reviewers' insightful suggestions and hope that the revised manuscript is now suitable for publication in ***PLOS ONE*** *Mental Health*.

Thank you for your time and consideration. We look forward to hearing from you.

| **Editor/Reviewer Comment** | **Author Comment** |
| --- | --- |
| At this time, please upload the minimal data set necessary to replicate your study's findings to a stable, public repository (such as figshare or Dryad) and provide us with the relevant URLs, DOIs, or accession numbers that may be used to access these data. For a list of recommended repositories and additional information on PLOS standards for data deposition, please see <https://journals.plos.org/plosone/s/recommended-repositories>. | Thank you! We have uploaded the data to Figshare. <https://doi.org/10.6084/m9.figshare.32885093> |
| Please ensure that your Ethics Statement is available in its entirety at the beginning of your Methods section, under a subheading 'Ethics Statement'. It must include:  i) The name(s) of the Institutional Review Board(s) or Ethics Committee(s)  ii) The approval number(s), or a statement that approval was granted by the named board(s)  iii) (for human participants or donors) - A statement that formal consent was obtained (must state whether verbal/written) OR the reason consent was not obtained (e.g., anonymity).  vi) (for child participants) - Please update your Ethics Statement with confirmation that written informed consent was obtained from the parent/guardian of each participant under 18 years of age.  If the reviewer comments include a recommendation to cite specific previously published works, please review and evaluate these publications to determine whether they are relevant and should be cited. There is no requirement to cite these works unless the editor has indicated otherwise. | We have created an Ethics Statement subsection at the beginning of the Methods section with the requested information. |
| Reviewer 1 |  |
| 1.The abstract states that the participants were "recruited through an undergraduate introductory psychology course in regular education". This is typically a convenience sample, which may have significant sample bias. Students who take the introductory psychology course may already be more sensitive to psychological issues, or the course may require participation in the research as a credit requirement, thereby affecting the motivation to participate and the authenticity of the responses. Moreover, all the participants come from the same university, which may limit the generalization of the conclusions to different institutions, majors, age groups, and non-undergraduate students. | Thank you for this comment. We have added a limitation to the Limitation section that acknowledges this. |
| 2. The statements such as "participation degree... is directly related", "higher sense of well-being... alleviates symptoms", and "mediating effect" used in the abstract are common in SEM, but cross-sectional data cannot support the causal direction. It is entirely possible that there is a reverse causality (i.e., college students with higher mental health levels are more actively involved in community activities) or that a third variable (such as personality traits, family support) simultaneously affects participation and psychological outcomes. Words like "influence" and "cause" should be used with caution. | We agree. We have tempered many of the statements in the manuscript to ensure we are not overstating our findings. For example:   - “This study examined the ~~impact~~ associations between…” - “The association between engagement and mental illness symptoms was statistically mediated by individual flourishing…” - “…the purpose of this study was to assess how community engagement across ecological levels (e.g., family, school, work, church) ~~influenced~~ is associated with individual flourishing…”   We have also rewritten the first limitation to better acknowledge this limitation. |
| 3. Only qualitative conclusions were provided in the abstract, without reporting any key statistical figures. It is necessary to appropriately report model fitting indicators, standardized coefficients of each path, standard errors, p-values, and confidence intervals, etc. | Thank you. We agree. As such we have revised the abstract to report the model fit, path coefficients, and significance levels for main findings. We also added quantitative information regarding the indirect associations. |
| Reviewer 2 |  |
| 1. There are few other questions to answer to define conclusion obtained in survey; such as Is "family engagement" psychologically equivalent to work engagement? Does absorption in one's family function the same way as absorption in work? Are vigor, dedication, and absorption meaningful indicators of church engagement? | Thank you for this comment. We agree that engagement in community domain is not necessarily identical to engag3ment in occupational settings. Our intention was to assess whether the engagement dimensions of vigor, dedication, and absorption could be meaningfully applied to these community domains. We selected the UWES-3 because it measures these general psychological states efficiently. To address this concern, we have added a statement to the limitation section. |
| 2. The manuscript needs a stronger justification for these adaptations. The authors should discuss prior research using adapted UWES measures, content validity considerations, and potential limitations of applying occupational engagement constructs to interpersonal and religious contexts. | Thank you for this suggestion. We have expanded the justification for adapting the UWES-3 by citing previous research extending engagement beyond occupational settings. We also added the conceptual and psychometric limitation across all the domain areas to the limitation section. |
| 3. In a sample where nearly all participants belong to the same religious tradition, church engagement may function differently than in more diverse populations. The authors should more explicitly acknowledge that church engagement may reflect cultural conformity, social integration, and institutional support, rather than purely religious participation. This distinction is important when interpreting findings. What do you think? | Yeah…we agree. We have added test to the discussion and limitations sections acknowledging that church engagement in this study may also be the result of broader social and community factors. We also note that homogeneous nature of the sample may limit generalizability of the findings. |
| 4. The manuscript introduces financial/material security as a second factor emerging from the flourishing measure. However, it is not clearly incorporated into the hypotheses, it is not adequately explained conceptually, and disappears from much of the Results and Discussion. Authors should consider why was this construct included? And What was its theoretical role? | Thank you. We agree the role of financial/material security was not explained well in the original manuscript. In response, we tweaked the introduction and measures sections to clarify that this dimension is important to within VanderWeele’s model of human flourishing. We also explain that financial/material security items emerged as distinct factors in the measurement model and were explored alongside individual flourishing. The purpose statement was revised in the introduction and discussion sections to broader human flourishing framework while the primary hypothesis remained focused on individual flourishing. |
| While revising your submission, we strongly recommend that you use PLOS’s NAAS tool (<https://ngplosjournals.pagemajik.ai/artanalysis>) to test your figure files. NAAS can convert your figure files to the TIFF file type and meet basic requirements (such as print size, resolution), or provide you with a report on issues that do not meet our requirements and that NAAS cannot fix. | Thank you. We have used this tool and resubmitted Figure 1. |
