# Supplementary material for: Associations between community engagement, human flourishing, and mental illness symptoms among college students
Source: PLOS Ment Health. 2026 Aug 17;3(8):e0000690. doi: 10.1371/journal.pmen.0000690 (PMC13480599; doi:10.1371/journal.pmen.0000690)
Supplement: Peer review history

# Peer review: Decision Letter 0

**DOI:** 10.1371/journal.pmen.0000690.r001

**Authors:** Wenxi Sun

10 Jun 2026

PMEN-D-26-00202

Associations between community engagement, individual flourishing, and mental illness symptoms among college students

PLOS Mental Health

Dear Dr. Carl L. Hanson,

Thank you for submitting your manuscript to PLOS Mental Health. After careful consideration, we feel that it has merit but does not fully meet PLOS Mental Health’s publication criteria as it currently stands. Therefore, we invite you to submit a revised version of the manuscript that addresses the points raised during the review process.

Please submit your revised manuscript by 20July2026. If you will need more time than this to complete your revisions, please reply to this message or contact the journal office at mentalhealth@plos.org. When you're ready to submit your revision, log on to https://www.editorialmanager.com/pmen/ and select the 'Submissions Needing Revision' folder to locate your manuscript file.

Please include the following items when submitting your revised manuscript:

- A letter that responds to each point raised by the editor and reviewer(s). You should upload this letter as a separate file labeled 'Response to Reviewers'.
- A marked-up copy of your manuscript that highlights changes made to the original version. You should upload this as a separate file labeled 'Revised Manuscript with Track Changes'.
- An unmarked version of your revised paper without tracked changes. You should upload this as a separate file labeled 'Manuscript'.

Guidelines for resubmitting your figure files are available below the reviewer comments at the end of this letter.

As the corresponding author, your ORCID iD is verified in the submission system and will appear in the published article. PLOS supports the use of ORCID, and we encourage all coauthors to register for an ORCID iD and use it as well. Please encourage your coauthors to verify their ORCID iD within the submission system before final acceptance, as unverified ORCID iDs will not appear in the published article. *Only*  the individual author can complete the verification step; PLOS staff *cannot*  verify ORCID iDs on behalf of authors.

We look forward to receiving your revised manuscript.

Kind regards,

Wenxi Sun, M.D.

Academic Editor

PLOS Mental Health

**Journal Requirements:**

1. Thank you for uploading your study's underlying data set. Unfortunately, the repository you have noted in your Data Availability statement does not qualify as an acceptable data repository according to PLOS's standards.

At this time, please upload the minimal data set necessary to replicate your study's findings to a stable, public repository (such as figshare or Dryad) and provide us with the relevant URLs, DOIs, or accession numbers that may be used to access these data. For a list of recommended repositories and additional information on PLOS standards for data deposition, please see https://journals.plos.org/plosone/s/recommended-repositories.

2. Please ensure that your Ethics Statement is available in its entirety at the beginning of your Methods section, under a subheading 'Ethics Statement'. It must include:

i) The name(s) of the Institutional Review Board(s) or Ethics Committee(s)

ii) The approval number(s), or a statement that approval was granted by the named board(s)

iii) (for human participants or donors) - A statement that formal consent was obtained (must state whether verbal/written) OR the reason consent was not obtained (e.g., anonymity).

vi) (for child participants) - Please update your Ethics Statement with confirmation that written informed consent was obtained from the parent/guardian of each participant under 18 years of age.

If the reviewer comments include a recommendation to cite specific previously published works, please review and evaluate these publications to determine whether they are relevant and should be cited. There is no requirement to cite these works unless the editor has indicated otherwise.

**Additional Editor Comments (if provided):**

I kindly request that the author carefully consider the reviewers’ comments and make the necessary revisions based on their feedback, as this is the only way to ensure the manuscript meets the journal’s publication standards.

[Note: HTML markup is below. Please do not edit.]

Reviewers' comments:

Reviewer's Responses to Questions

**Comments to the Author**

1. Does this manuscript meet PLOS Mental Health’s publication criteria?

Reviewer #1: Yes

Reviewer #2: Yes

**********

2. Has the statistical analysis been performed appropriately and rigorously?

Reviewer #1: Yes

Reviewer #2: I don't know

**********

3. Have the authors made all data underlying the findings in their manuscript fully available (please refer to the Data Availability Statement at the start of the manuscript PDF file)?

The PLOS Data policy

Reviewer #1: Yes

Reviewer #2: Yes

**********

4. Is the manuscript presented in an intelligible fashion and written in standard English?

Reviewer #1: Yes

Reviewer #2: Yes

**********

**Reviewer #1:**  This study examines how the participation of college students in various communities, such as family, school, work, and church, affects depression and anxiety symptoms through their sense of well-being. The research topic has strong practical significance, especially in the current context where the mental health problems of college students are increasingly prominent. The application of structural equation modeling also demonstrates appropriate methodological considerations. However, there are several issues that need to be revised or clarified in terms of sample representativeness, the clarity of measurement tools, the caution in causal inference, and the completeness of model result reporting.

1.The abstract states that the participants were "recruited through an undergraduate introductory psychology course in regular education". This is typically a convenience sample, which may have significant sample bias. Students who take the introductory psychology course may already be more sensitive to psychological issues, or the course may require participation in the research as a credit requirement, thereby affecting the motivation to participate and the authenticity of the responses. Moreover, all the participants come from the same university, which may limit the generalization of the conclusions to different institutions, majors, age groups, and non-undergraduate students.

2. The statements such as "participation degree... is directly related", "higher sense of well-being... alleviates symptoms", and "mediating effect" used in the abstract are common in SEM, but cross-sectional data cannot support the causal direction. It is entirely possible that there is a reverse causality (i.e., college students with higher mental health levels are more actively involved in community activities) or that a third variable (such as personality traits, family support) simultaneously affects participation and psychological outcomes. Words like "influence" and "cause" should be used with caution.

3. Only qualitative conclusions were provided in the abstract, without reporting any key statistical figures. It is necessary to appropriately report model fitting indicators, standardized coefficients of each path, standard errors, p-values, and confidence intervals, etc.

**Reviewer #2:**  The manuscript examines whether engagement across multiple community domains (family, peers, school, and church) is associated with flourishing and whether flourishing mediates associations between engagement and symptoms of depression and anxiety among college students. The integration of ecological systems theory and flourishing frameworks is a notable strength.

However, the manuscript currently doesn’t read like a journal article. I would encourage the authors to strengthen the conceptual model, provide more extensive psychometric evidence, and interpret findings with greater theoretical depth. In order to strengthen the manuscript, I have following comments for authors to consider:

1. There are few other questions to answer to define conclusion obtained in survey; such as Is "family engagement" psychologically equivalent to work engagement? Does absorption in one's family function the same way as absorption in work? Are vigor, dedication, and absorption meaningful indicators of church engagement?

2. The manuscript needs a stronger justification for these adaptations. The authors should discuss prior research using adapted UWES measures, content validity considerations, and potential limitations of applying occupational engagement constructs to interpersonal and religious contexts.

3. In a sample where nearly all participants belong to the same religious tradition, church engagement may function differently than in more diverse populations. The authors should more explicitly acknowledge that church engagement may reflect cultural conformity, social integration, and institutional support, rather than purely religious participation. This distinction is important when interpreting findings. What do you think?

4. The manuscript introduces financial/material security as a second factor emerging from the flourishing measure. However, it is not clearly incorporated into the hypotheses, it is not adequately explained conceptually, and disappears from much of the Results and Discussion. Authors should consider why was this construct included? And What was its theoretical role?

**********

what does this mean?). If published, this will include your full peer review and any attached files.

**Do you want your identity to be public for this peer review?**  If you choose “no”, your identity will remain anonymous but your review may still be made public.

For information about this choice, including consent withdrawal, please see our Privacy Policy

Reviewer #1: No

Reviewer #2: No

**********

[NOTE: If reviewer comments were submitted as an attachment file, they will be attached to this email and accessible via the submission site. Please log into your account, locate the manuscript record, and check for the action link "View Attachments". If this link does not appear, there are no attachment files.]

**Figure Resubmissions:**

# Author comment: Author response to Decision Letter 1

**DOI:** 10.1371/journal.pmen.0000690.r002

2 Jul 2026

Attachment Submitted filename: Response to Reviewers.docx

# Editor report: Decision Letter 1

**DOI:** 10.1371/journal.pmen.0000690.r003

**Authors:** Wenxi Sun

27 Jul 2026

Associations between community engagement, human flourishing, and mental illness symptoms among college students

PMEN-D-26-00202R1

Dear Carl L. Hanson,

We are pleased to inform you that your manuscript 'Associations between community engagement, human flourishing, and mental illness symptoms among college students' has been provisionally accepted for publication in PLOS Mental Health.

Before your manuscript can be formally accepted you will need to complete some formatting changes, which you will receive in a follow up email. A member of our team will be in touch with a set of requests.

Please note that your manuscript will not be scheduled for publication until you have made the required changes, so a swift response is appreciated.

IMPORTANT: The editorial review process is now complete. PLOS will only permit corrections to spelling, formatting or significant scientific errors from this point onwards. Requests for major changes, or any which affect the scientific understanding of your work, will cause delays to the publication date of your manuscript.

If your institution or institutions have a press office, please notify them about your upcoming paper to help maximize its impact. If they'll be preparing press materials, please inform our press team as soon as possible -- no later than 48 hours after receiving the formal acceptance. Your manuscript will remain under strict press embargo until 2 pm Eastern Time on the date of publication. For more information, please contact mentalhealth@plos.org.

Thank you again for supporting Open Access publishing; we are looking forward to publishing your work in PLOS Mental Health.

Best regards,

Wenxi Sun, M.D.

Academic Editor

PLOS Mental Health

***********************************************************

-

Reviewer Comments (if any, and for reference):
